# Fine-grained age-matching improves atrophy-based detection of mild cognitive impairment more than amyloid-negative reference subjects

**DOI:** 10.1016/j.nicl.2023.103508

**Published:** 2023-09-09

**Authors:** Nils Richter, Stefanie Brand, Nils Nellessen, Julian Dronse, Hannes Gramespacher, Maximilian H.T. Schmieschek, Gereon R. Fink, Juraj Kukolja, Oezguer A. Onur, for the Alzheimer's Disease Neuroimaging Initiative

**Affiliations:** a Cognitive Neuroscience, Institute of Neuroscience and Medicine (INM-3), Research Center Jülich, 52425 Jülich, Germany; b Department of Neurology, University Hospital Cologne and Faculty of Medicine, University of Cologne, 50937 Cologne, Germany; c Department of Neurology and Clinical Neurophysiology, Helios University Hospital Wuppertal, 42283 Wuppertal, Germany; d Faculty of Health, Witten/Herdecke University, 58448 Witten, Germany

**Keywords:** Gray matter, Alzheimer’s disease, MRI, Z-statistics, ADNI, Voxel-based-morphometry, CAT12, DARTEL

## Abstract

**Introduction:**

In clinical practice, differentiating between age-related gray matter (GM) atrophy and neurodegeneration-related atrophy at early disease stages, such as mild cognitive impairment (MCI), remains challenging. We hypothesized that fined-grained adjustment for age effects and using amyloid-negative reference subjects could increase classification accuracy.

**Methods:**

T1-weighted magnetic resonance imaging (MRI) data of 131 cognitively normal (CN) individuals and 91 patients with MCI from the Alzheimer’s disease neuroimaging initiative (ADNI) characterized concerning amyloid status, as well as 19 CN individuals and 19 MCI patients from an independent validation sample were segmented, spatially normalized and analyzed in the framework of voxel-based morphometry (VBM). For each participant, statistical maps of GM atrophy were computed as the deviation from the GM of CN reference groups at the voxel level. CN reference groups composed with different degrees of age-matching, and mixed and strictly amyloid-negative CN reference groups were examined regarding their effect on the accuracy in distinguishing between CN and MCI. Furthermore, the effects of spatial smoothing and atrophy threshold were assessed.

**Results:**

Approaches with a specific reference group for each age significantly outperformed all other age-adjustment strategies with a maximum area under the curve of 1.0 in the ADNI sample and 0.985 in the validation sample. Accounting for age in a regression-based approach improved classification accuracy over that of a single CN reference group in the age range of the patient sample. Using strictly amyloid-negative reference groups improved classification accuracy only when age was not considered.

**Conclusion:**

Our results demonstrate that VBM can differentiate between age-related and MCI-associated atrophy with high accuracy. Crucially, age-specific reference groups significantly increased accuracy, more so than regression-based approaches and using amyloid-negative reference groups.

## Highlights

• Z-statistics of gray matter can differentiate atrophy related to mild cognitive impairment and that observed in cognitively normal aging with great accuracy.
• Use of reference groups closely matched to the age of the examined individual outperform approaches commonly used today.
• Amyloid status of the reference groups does not affect classification accuracy if age is adequately controlled.

## Graphical abstract

**Figure:**
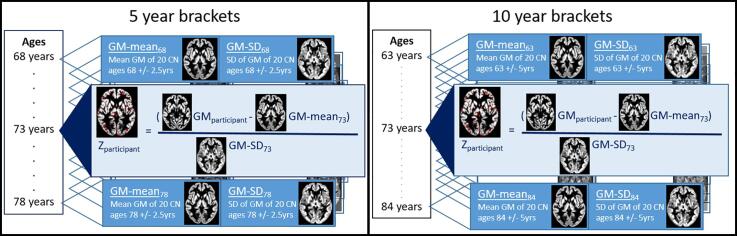

## 1 Introduction

Mild cognitive impairment (MCI), characterized by unimpaired activities of daily living in the presence of objectifiable cognitive deficits [Albert et al., 2011], often precedes dementia in the course of Alzheimer’s disease (AD). MCI is associated with an increased risk of progression to dementia due to AD [Lombardi et al., 2020, Mitchell and Shiri-Feshki, 2009], but it can also be caused by other neurodegenerative diseases, vascular lesions, medication side effects, other medical conditions, e.g., depression [Lopez et al., 2003].

Cerebral imaging plays a central role in the diagnostic work-up of MCI to rule out underlying structural lesions and to detect cerebral atrophy to guide further diagnostic steps. However, since neurodegenerative changes overlap with age-related atrophy [Franke et al., 2010, Raji et al., 2009], it remains challenging to differentiate between the two.

Different approaches have been taken to standardize the quantification of brain atrophy. Semiquantitative rating scales of global and regional cerebral atrophy allow a quick assessment [Koedam et al., 2011, Pasquier et al., 1996, Scheltens et al., 1992] but show inter-rater variability [Pasquier et al., 1996, Scheltens et al., 1992, Scheltens et al., 1995].

An alternative is quantifying the deviation of brain and tissue volumes from the mean of a control sample, as is common practice in evaluating cerebral glucose metabolism measured using positron emission tomography (PET) [Minoshima et al., 1995]. In the case of MRI-based atrophy measurements, a patient’s high-resolution T1-weighted image is automatically segmented into tissue classes and spatially normalized to a reference template. The difference between the patient’s gray matter (GM) and the mean GM of a control group is then computed and expressed as multiples of the standard deviation of the control group, i.e., Z-statistics [Matsuda et al., 2012]. It is possible to differentiate between cognitively normal (CN) and AD dementia with high accuracy using various indices. However, the classification accuracy is considerably lower when distinguishing between CN and MCI [Li et al., 2019, Waragai et al., 2014].

The reference group in this approach is age-matched to the patient sample but typically with an age range of around 30 years [Hirata et al., 2005, Komatsu et al., 2018, Matsuda et al., 2012, Matsunari et al., 2007, Tateno et al., 2015, Waragai et al., 2014], inducing a considerable bias if a patient’s age is close to the extremes of the reference sample. Alternatively, a regression-based approach can be used to account for confounds such as age. Here, the confound is regressed upon a reference sample, and the resulting parameter estimates are used to compute an expected GM volume for the individual patient’s level of that confound. The expected GM volume is then compared to the actual GM volume of the patient [Alzheimer’s Disease Neuroimaging Initiative et al., 2015, Hedderich et al., 2020, Hedderich et al., 2022, Mühlau et al., 2009]. However, this approach assumes a linear relationship between the measure of interest and confounds, which, in the case of age, is not necessarily valid [Dima et al., 2022, Fjell et al., 2013, Hedman et al., 2012].

Another aspect is that control samples typically are not selected according to their amyloid status [Alzheimer’s Disease Neuroimaging Initiative et al., 2015, Hedderich et al., 2020, Hedderich et al., 2022, Hirata et al., 2005, Matsuda et al., 2012, Waragai et al., 2014]. However, there is evidence that even CN amyloid-positive individuals may exhibit reduced GM volumes compared to amyloid-negative individuals [Becker et al., 2011, Harrison et al., 2021, Whitwell et al., 2013]. An amyloid-negative reference group without amyloid-related atrophy could thus be more sensitive to disease-related atrophy.

We hypothesized that optimizing the reference group could improve MR-based differentiation between CN and MCI patients. Specifically, we hypothesized that an approach with amyloid-negative reference groups closely matched to an individual patient’s age would be more sensitive to neurodegenerative changes in GM volume, resulting in greater classification accuracy, even compared to regression-based approaches. Using structural MRI, as well as amyloid-PET data of CN participants and patients with MCI from the Alzheimer’s Disease Neuroimaging Initiative (ADNI) database (adni.loni.ucla.edu) and an independent sample acquired in our center, we investigated the effects of different types of reference groups on the utility of Z-statics-based atrophy quantification in differentiating between CN and MCI. Furthermore, we examined the effects of atrophy thresholds, the extent of spatial smoothing, and region of interest (ROI) on classification accuracy.

## 2 Methods

Data used to prepare this study were obtained from the Alzheimer’s Disease Neuroimaging Initiative (ADNI) database (adni.loni.usc.edu). The ADNI was launched in 2003 as a public–private partnership led by Principal Investigator Michael W. Weiner, MD. The primary goal of ADNI has been to test whether serial magnetic resonance imaging (MRI), positron emission tomography (PET), other biological markers, and clinical and neuropsychological assessment can be combined to measure the progression of mild cognitive impairment (MCI) and early Alzheimer’s disease (AD).

### 2.1 Subjects

Structural MRI and (AV45) amyloid-PET scans of CN individuals and participants with MCI were retrieved from the ADNI-GO and the ADNI-2 phases. Detailed documentation of the inclusion criteria and diagnostic categories can be found on the ADNI website (https://adni.loni.usc.edu/methods/documents/).

In short, CN was defined by an MMSE greater than 23 points, a Clinical Dementia Rating of 0, and neuropsychological performance within normal ranges. MCI was defined as an MMSE greater than 23 points, a CDR of 0.5, a subjective memory concern reported by the patient, caregiver, or treating clinician, a memory loss objectified using the education-adjusted delayed-recall performance on the Wechsler Memory Scale Logical Memory II, and preserved activities of daily living.

For each participant, the T_1_ scan performed closest to an amyloid-PET scan was selected and classified as CN or MCI based on the neuropsychological assessment with the shortest delay from the MRI scan. Participants with cerebral infarcts or significant depressive symptoms, indicated by a GDS score greater than 5, were not included in the analysis. Furthermore, seven CN participants and two MCI patients were excluded because of confluent white matter lesions corresponding to Fazekas grade 3 [Fazekas et al., 1993], and four CN and one MCI participant had to be excluded due to image processing failures. The resulting final data set included a total of 141 cognitively normal individuals and 91 participants with MCI. The average time between PET and MRI measurements was 28.31 ± 28.46 days.

### 2.2 Confirmatory sample

Findings were validated on an independent sample of CN and MCI participants acquired in our center as part of a study approved by the ethics committee of the University of Cologne's medical faculty. All participants gave written informed consent. In this sample, CN was defined by an MMSE greater than 23 points and unimpaired performance on neuropsychological tests of memory, language, and executive function. MCI was defined as an MMSE greater than 23 points, a subjective memory concern reported by the patient, caregiver, or treating clinician, memory loss objectified using the age-adjusted delayed-recall performance on the Wechsler Memory Scale Logical Memory II, and preserved activities of daily living, based on information provided by the spouse or a caregiver. This data set consisted of 19 CN individuals and 19 MCI patients. MCI patients presented with a predominantly amnestic phenotype, and 18/19 of patients had positive CSF biomarkers or Amyloid-PET indicative of Alzheimer’s pathology as previously described [Conwell et al., 2018, Richter et al., 2020].

### 2.3 PET and MRI acquisition

ADNI-GO/-2 MRI data were acquired on 3 T MRI scanners by Siemens, Philips, and General Electric Healthcare. The present study used the scanner-specific 3D sagittal T_1_-weighted magnetization-prepared rapid gradient-echo (MPRAGE) sequences. ADNI’s original MPRAGE sequences undergo standardized image correction steps during preprocessing to increase signal uniformity across different scanner types and trial centers.

AV45-PET data were also acquired on different scanners at the different trial centers. Therefore, the PET data in the ADNI study also undergo standardized preprocessing steps to increase signal uniformity across centers. The imaging protocols used at the different trial centers are described in detail on the ADNI website (https://adni.loni.usc.edu/data-samples/).

T_1_-weighted MPRAGE images for the confirmatory sample were acquired using a 3 T MAGNETOM Trio (Siemens, Erlangen, Germany) with a custom build BrainPET insert in the bore of the magnet using both a transmit-receive and 8-channel receive coil. The scan parameters were: TR = 2250 ms, TE = 3.03 ms, FA = 9°, FOV = 256 × 256 mm^2^, matrix = 256 × 256, voxel resolution = 1 mm isotropic, 176 sagittal slices, no gap, interleaved, scan time = 5 min and 14 s. Vacuum cushions were used to reduce head motion. Automated and manual shimming was applied before data acquisition to account for field inhomogeneities resulting from the BrainPET insert.

### 2.4 MRI data processing – Voxel-based morphometry (VBM)

MRI data were processed using statistical parametric mapping (SPM12, Wellcome Trust Center for Neuroimaging) with the computational anatomy toolbox (CAT12, https://www.neuro.uni-jena.de/cat/) implemented in MatLab R2012b (MathWorks, Natick, MA, USA).

Images were bias-corrected and automatically segmented into GM, white matter (WM), and cerebrospinal fluid (CSF). The GM segment underwent visual inspection for misclassification of tissue. GM maps of three CN and one MCI participant had to be excluded from further analyses because of tissue classification errors.

Tissue maps resulting from the segmentation were high-dimensionally warped to a study-specific template using diffeomorphic anatomical registration through exponentiated Lie algebra (DARTEL) [Ashburner, 2007]. The template was generated from the T_1_ images of 34 amyloid-positive and 34 amyloid-negative CN patients of the ADNI sample, which were matched according to age and gender. The GM partitions were warped to the template space and modulated for the nonlinear normalization only to preserve tissue concentrations while accounting for differences in TIV. Resulting GM maps were smoothed with Gaussian kernels of 2 mm, 4 mm, and 8 mm full width at half maximum (FWHM) for subsequent analyses.

### 2.5 Regions of interest

Atrophy was assessed at the level of total GM and cortical GM in the individual cerebral lobes defined using the Montreal Neurological Institute (MNI) atlas [Mazziotta et al., 2001], as well as a medial temporal lobe (MTL) ROI, including the hippocampus, the amygdala, the parahippocampal, and the temporal fusiform gyri, defined using the Harvard-Oxford atlas [Desikan et al., 2006].

### 2.6 Amyloid status

Amyloid status in the ADNI sample was defined based on the standard uptake value ratios (SUVR) of florbetapir, as made available from ADNI. Briefly, in the framework of ADNI, preprocessed florbetapir scans (https://adni.loni.usc.edu/methods/pet-analysis-method/pet-analysis/) were coregistered to the participants’ T_1_ scans, which were segmented and parcellated into ROI using the software Freesurfer (https://surfer.-nmr.mgh.harvard.edu/, version 5.3.0). The SUVR for each participant was then obtained by dividing the mean florbetapir uptake in a set of cortical ROI by the florbetapir uptake in the whole cerebellum (white and gray matter) [Landau et al., 2012]. Participants with a florbetapir SUVR greater than 1.11 were considered amyloid-positive, and those with a florbetapir SUVR < 1.11 were considered amyloid-negative [Clark, 2011, Joshi et al., 2012]. Amyloid status in the confirmatory sample was defined based on cerebro-spinal fluid (CSF) biomarkers or clinical amyloid-PET.

### 2.7 Statistics

Data were tested for normality of distribution using the Shapiro-Wilk-Test. Group comparisons of normally distributed data were performed using independent samples T-tests and the Wilcoxon rank-sum test for non-normally distributed data. Gender distribution was assessed using the Chi-Square-Test. Statistical analyses were performed with the software R (Version 3.6.3, https://cran.r-project.org/). Receiver operating characteristic (ROC) analyses were within R using the package “pROC”. Image arithmetics and spatial smoothing were performed with modules of the FSL software package (FMRIB’s Software Library, Version 5.0, https://www.fmrib.ox.ac.uk/fsl).

### 2.8 Definition of atrophy

Atrophy was operationalized for each participant as the deviation of GM volume from the mean of a reference group: GM maps of participants were transformed to voxel-wise Z-statistics by subtracting the mean of a reference group and dividing it by the standard deviation of that reference group. As the central element of this investigation, the effect of using different reference groups was analyzed (see below). The voxel-wise Z-maps were thresholded at different Z-levels (-2.5, −3.5, and −4.5), and the number of subthreshold voxels was assessed in each ROI. For each ROI, the number of subthreshold voxels was entered in a ROC analysis to determine the accuracy with which the participants could be classified as CN or MCI patients.

### 2.9 Types of reference groups

**Fig. 1:**
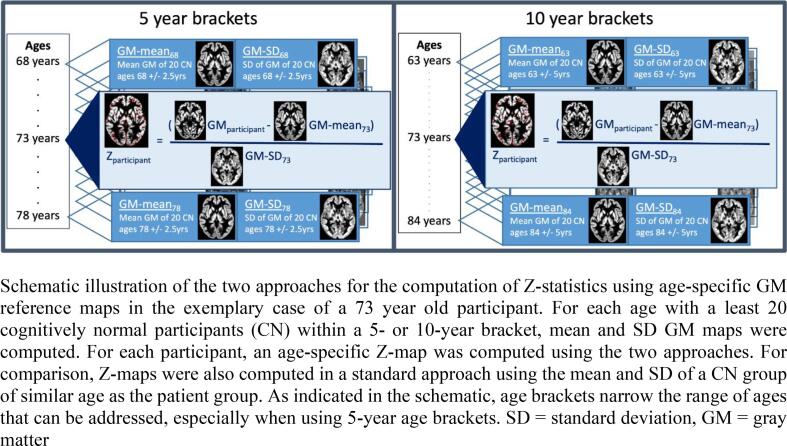
Schematic illustration of the two approaches for the computation of Z-statistics using age-specific GM reference maps in the exemplary case of a 73 year old participant. For each age with a least 20 cognitively normal participants (CN) within a 5- or 10-year bracket, mean and SD GM maps were computed. For each participant, an age-specific Z-map was computed using the two approaches. For comparison, Z-maps were also computed in a standard approach using the mean and SD of a CN group of similar age as the patient group. As indicated in the schematic, age brackets narrow the range of ages that can be addressed, especially when using 5-year age brackets. SD = standard deviation, GM = gray matter.

Age effects were investigated by comparing the performance of two approaches using different age-specific reference groups to the ‘standard’ approach of using one reference group consisting of CN participants in the same age range as the whole patient group [Hirata et al., 2005, Matsuda, 2016] and a regression-based approach explained below. In the first type of age-specific approach, mean and standard deviation GM maps were computed from 20 CN participants whose age deviated less than five years from the respective age. The second type of age-specific approach used reference groups of 20 CN participants whose age deviated<2.5 years from the age investigated (Fig. 1). Z-statistics were computed for each participant, comparing them with the mean of the reference group corresponding to their age. In the ‘standard’ approach, the mean and standard deviation of the 141 CN participants from the ADNI sample were used.

For the two age-specific approaches, Z-statistics could not be computed for all participants due to the normal age distribution with the consequence of having<20 CN to compute mean and standard deviation images at the lower and upper extremes of the age range. Using all 141 CN participants, reference groups of 20 participants with ages ± 2.5 years of the participant could be generated for ages 66 to 80 years and ± 5 years of the participant for ages 63 to 82 years.

In order to assess the relevance of amyloid status for classification accuracy, the same analyses were also performed using only the 97 amyloid-negative CN participants to form the reference groups. Using only amyloid-negative CN, reference groups with ages within 2.5 years of the participant were generated for ages 68 to 78 and within five years of the participant for ages 63 to 84.

The difference in the number of subthreshold voxels between groups was assessed using Wilcoxon rank-sum tests because of their non-normal distribution. Differences were deemed significant at a p < 0.0001, corresponding to a Bonferroni-corrected p = 0.0216.

### 2.10 ROC analyses

ROC analyses were performed to determine the parameters that distinguish CN and MCI with the highest accuracy. Specifically, we examined the effect of smoothing (unsmoothed, Gaussian kernels of 2 mm, 4 mm, and 8 mm FWHM), Z-threshold, amyloid stats, and region of interest. The statistical significance of the difference in AUC was assessed using DeLong’s Test as implemented in the “R”-package ‘pROC’. The same analyses were applied to an independent validation sample to validate our observations for the optimal parameters.

### 2.11 W-Scores

A number of publications have reported on the use of W-scores to adjust for covariates such as age using linear regression. We have included additional analyses to compare the performance of this approach to the use of age-specific references. For details please see the Supplementary Materials.

## 3 Results

### 3.1 Demographics

MCI patients in sample 1 (ADNI) were younger than CN (MCI 70.84 ± 7.50 years, CN 73.45 ± 5.75 years, p = 0.004). In the confirmatory sample, however, MCI patients were older than CN (MCI 72.78 ± 3.91 years, CN 68.53 ± 3.04 years, p = 0.001) (Table 1, Table 2).

**Table 1:** Demographic and neuropsychological characteristics of the ADNI sample.

	Cognitively normal (CN)				Mild cognitive impairment (MCI)				Group difference
	Amyloid- negative (n = 97, 76%)	Amyloid-positive (n = 34, 24%)	Group Difference p-value	Total (n = 131)	Amyloid-negative (n = 49, 54%)	Amyloid-positive (n = 42, 46%)	Group difference p-value	Total (n = 91)	CN vs. MCI
	Mean (SD)	Mean (SD)		Mean (SD)	Mean (SD)	Mean (SD)		Mean (SD)	p-value
Age	72.78 (5.63)	75.37 (5.74)	**0.023**	73.45 (5.75)	69.42 (7.64)	72.49 (7.06)	0.051	70.84 (7.50)	**0.004**
Gender (male/female)	50/48	13/21	0.198	63/69	24/25	24/18	0.437	48/43	0.815
Education (years)	16.62 (2.60)	16.03 (2.39)	0.147	16.47 (2.55)	16.96 (2.16)	16.55 (3.05)	0.731	16.77 (2.60)	0.313
MMSE	29.07 (1.27)	28.79 (1.25)	0.141	29.00 (1.27)	28.63 (1.42)	28.07 (1.79)	0.149	28.37 (1.62)	**0.003**
LM-DR	13.79 (3.15)	12.91 (2.95)	0.153	13.56 (3.11)	8.8 (1.77)	8.36 (2.09)	0.399	8.59 (1.93)	**< 0.001**
TMT-A	31.99 (10.72)	38.91 (10.81)	**< 0.001**	33.78 (11.08)	33.65 (11.73)	34.95 (9.37)	0.229	34.25 (10.67)	0.664
TMT-B	77.28 (43.13) ^1^	91.47 (39.74)	**0.007**	80.96 (42.42) ^1^	81.13 (35.68) ^2^	91.07 (36.97)	0.115	85.82 (36.43) ^2^	0.081

Except for age, data were not normally distributed. Group differences were computed with T-Tests or Wilcoxon-Tests as appropriate. The Chi-Square-Test was used to assess differences between gender distributions. Bold font indicates statistical significance at p < 0.05 (two-sided). SD = standard deviation; MMSE = Mini-Mental-Status-Exam; LM-DR = Logical Memory Delayed Recall; TMT-A = Trail Making Test Part A (Seconds); TMT-B = Trail Making Test Part B (Seconds). 1 = missing data for one participant. ^2^ = missing data for two participants.

**Table 2:** Demographic and neuropsychological characteristics of the validation sample.

	CN (n = 19)	MCI (n = 19)	CN vs. MCI
	Mean (SD)	Mean (SD)	p-value
Age	68.53 (3.04)	72.78 (3.91)	**0.001**
Gender (male/female)	14/5	11/8	0.305
Education (years)	14.16 (3.99)	13.11 (4.47)	0.418
MMSE	29.05 (1.31)	25.79 (1.27)	**< 0.001**
LM-DR	25.42 (6.22)	3.16 (3.25)	**< 0.001**
TMT-A	33.16 (11.03)	51.99 (21.60)	**0.002**
TMT-B	82.00 (29.71)	177.99 (96.88)	**< 0.001**

Except for age, data were not normally distributed. Group differences were computed with T-Tests or Wilcoxon-Tests as appropriate. The Chi-Square-Test was used to assess differences between gender distributions. Bold font indicates statistical significance at p < 0.05 (two-sided). CN = cognitively normal, MCI = mild cognitive impairment, SD = standard deviation; MMSE = Mini-Mental-Status-Exam, LM-DR = Logical Memory Delayed Recall, TMT-A = Trail Making Test Part A (Seconds), TMT-B = Trail Making Test Part B (Seconds).

MCI patients performed poorer than CN on the MMSE and the LM-DR in both samples and on the TMT in the validation sample. Interestingly, while CN and MCI of sample 1 did not differ concerning performance on the TMT, amyloid-positive CN performed worse on the TMT-A (CN amyloid-positive = 38.91 s ± 10.81 s, CN amyloid-negative = 31.99 s ± 10.72, p < 0.001) and B than amyloid-negative CN (CN amyloid-positive = 91.47 s ± 39.47 s, CN amyloid-negative = 77.28 s ± 43.13, p = 0.007; Table 1).

In sample 1 (ADNI), 34 of 141 CN (24%) and 42 of 91 MCI participants (46%) were amyloid-positive, while the majority of MCI participants in the validation sample (18 of 19) had CSF-biomarkers or amyloid-PET indicative of AD pathology. These biomarkers of AD pathology had not been assessed in the CN of the confirmatory sample.

There was no difference between CN and MCI groups with respect to gender distribution or level of education in either sample.

### 3.2 Group differences in the number of subthreshold voxels

Using the standard reference group, significant differences in the number of subthreshold voxels between CN and MCI were observed in frontal, temporal, parietal, and total GM at Z-thresholds of −3.5 and −4.5 using no smoothing and in frontal and total GM at 2 mm smoothing. Using age-specific reference groups, significant differences in the number of subthreshold voxels were observed for all ROI, Z-thresholds, and degrees of smoothing, except for MTL and lobar GM at a Z-threshold of −4.5 with 8 mm smoothing. There were no significant differences between the two types of age-specific reference groups concerning the number of subthreshold voxels (Table 3).

**Table 3:** Number of subthreshold voxels for CN and MCI determined using age-specific reference groups and the standard approach.

Standard approach (reference group of 141 CN)																	
Smoothing kernel		0 mm				2 mm				4 mm				8 mm			
Group		CN		MCI		CN		MCI		CN		MCI		CN		MCI	
ROI	Z	Mean	SD	Mean	SD	Mean	SD	Mean	SD	Mean	SD	Mean	SD	Mean	SD	Mean	SD
Total GM	−2.5	1770	906	2368	2421	1438	939	2069	2867	1203	1169	2015	4156	1128	1768	2406	7103
	−3.5	118	103	270	477	77	98	205	470	59	125	183	554	49	179	226	838
	−4.5	**5**	**11**	**33**	**100**	**3**	**9**	**22**	**85**	2	9	18	79	2	13	12	63
MTL	−2.5	119	186	309	530	99	198	311	597	90	237	351	744	95	325	466	1105
	−3.5	15	50	73	183	11	50	68	192	8	50	71	219	5	43	92	306
	−4.5	**1**	**7**	**17**	**66**	1	7	15	66	0	4	13	65	0	0	10	57
Frontal cortex	−2.5	521	250	705	615	410	274	602	836	320	403	568	1532	319	843	672	3128
	−3.5	**33**	**24**	**67**	**85**	**18**	**20**	**44**	**92**	10	20	34	156	4	32	47	344
	−4.5	**2**	**3**	**6**	**10**	1	2	2	5	0	0	1	4	0	0	0	3
Temporal cortex	−2.5	364	301	671	933	276	324	613	1067	241	419	685	1457	264	623	998	2490
	−3.5	32	64	112	246	21	65	94	249	19	86	96	287	21	132	135	433
	−4.5	**2**	**9**	**21**	**72**	1	8	17	71	1	7	15	72	2	13	11	62
Parietal cortex	−2.5	271	158	333	248	243	185	306	296	197	222	278	434	128	282	238	663
	−3.5	**14**	**17**	**25**	**30**	9	21	17	28	6	27	12	34	3	24	7	50
	−4.5	0	1	1	2	0	1	0	1	0	3	0	0	0	0	0	0
Occipital cortex	−2.5	158	111	189	146	131	116	160	168	106	157	139	264	88	217	159	488
	−3.5	7	9	12	13	5	10	8	13	5	18	6	20	5	31	8	47
	−4.5	0	0	0	1	0	0	0	1	0	0	0	0	0	2	0	0
10-year brackets (age-specific reference groups of 20 CN all within 5 years of the participant)																	
Smoothing kernel		0 mm				2 mm				4 mm				8 mm			
Group		CN		MCI		CN		MCI		CN		MCI		CN		MCI	
ROI	Z	Mean	SD	Mean	SD	Mean	SD	Mean	SD	Mean	SD	Mean	SD	Mean	SD	Mean	SD
Total GM	−2.5	**1206**	**617**	**4025**	**2116**	**946**	**635**	**3569**	**2501**	**774**	**805**	**3371**	**3597**	**740**	**1337**	**3577**	**6387**
	−3.5	**10**	**10**	**761**	**667**	**5**	**7**	**579**	**714**	**3**	**7**	**472**	**918**	**2**	**12**	**473**	**1470**
	−4.5	**0**	**0**	**174**	**262**	0	0	122	263	**0**	**0**	**98**	**304**	**0**	**0**	**93**	**382**
MTL	−2.5	**83**	**132**	**421**	**561**	**68**	**141**	**418**	**625**	**61**	**170**	**453**	**751**	**72**	**262**	**547**	**1088**
	−3.5	**1**	**5**	**119**	**248**	**1**	**4**	**109**	**262**	**0**	**1**	**108**	**295**	**0**	**1**	**116**	**360**
	−4.5	**0**	**0**	**41**	**135**	**0**	**0**	**37**	**140**	**0**	**0**	**35**	**150**	0	0	32	155
Frontal cortex	−2.5	**359**	**178**	**1196**	**507**	**268**	**182**	**1031**	**658**	**198**	**230**	**937**	**1090**	**174**	**435**	**977**	**2271**
	−3.5	**3**	**4**	**210**	**120**	**1**	**3**	**149**	**135**	**0**	**2**	**104**	**213**	**0**	**1**	**101**	**423**
	−4.5	**0**	**0**	**42**	**33**	**0**	**0**	**23**	**32**	**0**	**0**	**14**	**43**	0	0	13	76
Temporal cortex	−2.5	**245**	**185**	**993**	**950**	**181**	**197**	**903**	**1103**	**156**	**263**	**933**	**1478**	**186**	**453**	**1151**	**2422**
	−3.5	**2**	**6**	**220**	**345**	**1**	**4**	**177**	**371**	**1**	**3**	**173**	**474**	**1**	**11**	**200**	**689**
	−4.5	**0**	**0**	**60**	**153**	**0**	**0**	**48**	**160**	**0**	**0**	**47**	**191**	**0**	**0**	**50**	**233**
Parietal cortex	−2.5	**194**	**99**	**629**	**224**	**162**	**112**	**580**	**275**	**126**	**144**	**514**	**407**	**89**	**220**	**427**	**711**
	−3.5	**1**	**2**	**100**	**51**	**1**	**2**	**75**	**53**	**0**	**1**	**51**	**66**	**0**	**2**	**35**	**139**
	−4.5	**0**	**0**	**18**	**13**	**0**	**0**	**11**	**12**	**0**	**0**	**6**	**11**	0	0	2	15
Occipital cortex	−2.5	**107**	**67**	**401**	**210**	**86**	**70**	**362**	**254**	**70**	**105**	**340**	**414**	**64**	**214**	**383**	**834**
	−3.5	**0**	**1**	**62**	**45**	**0**	**1**	**47**	**49**	**0**	**1**	**36**	**73**	**0**	**0**	**45**	**167**
	−4.5	**0**	**0**	**10**	**11**	**0**	**0**	**7**	**11**	**0**	**0**	**5**	**17**	0	0	6	30
5-year brackets (age-specific reference groups of 20 CN all within 2.5 years of the participant)																	
Smoothing kernel		0 mm				2 mm				4 mm				8 mm			
Group		CN		MCI		CN		MCI		CN		MCI		CN		MCI	
ROI	Z	Mean	SD	Mean	SD	Mean	SD	Mean	SD	Mean	SD	Mean	SD	Mean	SD	Mean	SD
Total GM	−2.5	**1264**	**648**	**3916**	**2235**	**1001**	**667**	**3433**	**2671**	**834**	**850**	**3186**	**3916**	**829**	**1435**	**3375**	**7160**
	−3.5	**11**	**11**	**738**	**701**	**5**	**8**	**550**	**759**	**3**	**8**	**432**	**1000**	**2**	**13**	**451**	**1663**
	−4.5	**0**	**0**	**167**	**280**	**0**	**0**	**112**	**282**	**0**	**0**	**85**	**330**	**0**	**0**	**82**	**422**
MTL	−2.5	**88**	**142**	**384**	**564**	**72**	**151**	**378**	**627**	**65**	**181**	**407**	**755**	**76**	**277**	**501**	**1136**
	−3.5	**1**	**6**	**112**	**256**	**1**	**4**	**102**	**271**	**0**	**1**	**100**	**307**	**0**	**1**	**112**	**383**
	−4.5	**0**	**0**	**40**	**144**	**0**	**0**	**36**	**150**	**0**	**0**	**34**	**162**	0	0	32	172
Frontal cortex	−2.5	**365**	**183**	**1206**	**554**	**274**	**186**	**1046**	**723**	**206**	**237**	**948**	**1218**	**193**	**468**	**1010**	**2591**
	−3.5	**4**	**4**	**220**	**134**	**1**	**3**	**159**	**153**	**0**	**2**	**116**	**245**	**0**	**1**	**119**	**491**
	−4.5	**0**	**0**	**46**	**37**	**0**	**0**	**27**	**36**	**0**	**0**	**17**	**50**	0	0	16	88
Temporal cortex	−2.5	**257**	**197**	**936**	**988**	**193**	**209**	**843**	**1156**	**169**	**281**	**871**	**1580**	**206**	**487**	**1102**	**2676**
	−3.5	**3**	**6**	**212**	**366**	**1**	**4**	**169**	**393**	**1**	**4**	**162**	**502**	**1**	**13**	**202**	**759**
	−4.5	**0**	**0**	**60**	**166**	**0**	**0**	**46**	**173**	**0**	**0**	**45**	**206**	0	0	48	254
Parietal cortex	−2.5	**197**	**98**	**636**	**241**	**163**	**106**	**586**	**292**	**126**	**133**	**514**	**427**	**92**	**226**	**406**	**723**
	−3.5	**1**	**2**	**104**	**56**	**1**	**2**	**79**	**57**	**0**	**1**	**52**	**68**	**0**	**2**	**40**	**160**
	−4.5	**0**	**0**	**19**	**13**	**0**	**0**	**11**	**12**	**0**	**0**	**5**	**10**	0	0	3	18
Occipital cortex	−2.5	**110**	**68**	**395**	**225**	**89**	**72**	**355**	**278**	**73**	**110**	**334**	**464**	**70**	**230**	**391**	**939**
	−3.5	**1**	**1**	**62**	**46**	**0**	**1**	**47**	**53**	**0**	**1**	**37**	**82**	**0**	**0**	**48**	**192**
	−4.5	**0**	**0**	**10**	**11**	**0**	**0**	**7**	**12**	**0**	**0**	**6**	**19**	0	0	8	38

Group differences were computed as Wilcoxon-Tests. Bold font and gray background indicate a significant group difference at p < 0.0001. ROI = region of interest, Z = Z-threshold, MCI = mild cognitive impairment, CN = cognitively normal, SD = standard deviation, GM = gray matter, MTL = medial temporal lobe. The smoothing kernel is reported in mm of full width at half-maximum.

### 3.3 Classification accuracy based on the number of subthreshold voxels

ROC analyses revealed an optimal separation between CN and MCI using age-specific reference groups. Diagnostic accuracy was substantially higher when using the 10-year bracket approach than the standard reference group, reaching an AUC of 1 for temporal, parietal, and total GM when using 2 mm or no smoothing kernels. Using 5-year brackets did not increase diagnostic accuracy compared to 10-year brackets. The greatest AUC achieved using the standard approach was 0.731 for total GM without spatial smoothing, which is significantly poorer than the accuracies achieved for both age-specific approaches under those conditions (DeLong’s Tests, p < 0.001 compared to the age-specific approaches). Diagnostic accuracy increased with decreasing Z-thresholds, with the greatest accuracy generally seen around a Z-threshold of −3.5 (Table 4). Lowering the Z-threshold to −4.5 resulted in a decrease in AUC in some areas. The six ROIs did not differ significantly (all DeLong’s Tests, p greater than 0.05) for AUC at optimal conditions (0 or 2 mm smoothing, Z-threshold = -3.5). However, the AUC was greater in all other ROIs than in the MTL (for example compared to total GM at 0 mm and Z-threshold = -3.5, AUC_MTL_ = 0.985, AUC_total GM_ = 1, DeLong’s-Test p = 0.0263).

**Table 4:** Areas under the curve for the comparison of MCI and CN determined using different types of references.

ROI = region of interest, AUC = area under the curve, Z = Z-threshold, GM = gray matter, MTL = medial temporal lobe, CN = cognitively normal. The smoothing kernel is reported in mm full width at half-maximum.

### 3.4 Effect of amyloid status

When including only amyloid-negative CN in the reference groups, the greatest AUC for the standard reference group without age brackets was 0.825 for total GM (Table 5). The AUC for the amyloid-negative reference group without age brackets was greater than for the reference group without age brackets, including amyloid-negative and positive CN for 2 mm or no smoothing kernels. The most remarkable difference was observed for parietal GM at a Z-threshold of −4.5, but this difference was barely significant (AUC amyloid-negative CN reference group = 0.689, AUC amyloid-negative and –positive reference group = 0.593, DeLong’s Test p = 0.039). Using amyloid-negative references and age-specific reference groups did not improve AUC values (Table 4, Table 5).

**Table 5:** Areas under the curve for comparing MCI and CN, determined using different amyloid-negative references.

ROI = region of interest, AUC = area under the curve, Z = Z-threshold, GM = gray matter, MTL = medial temporal lobe, CN = cognitively normal. The smoothing kernel is reported in mm full width at half-maximum.

### 3.5 Optimal cut-off values

The highest accuracy for distinguishing between CN and MCI was achieved using age-specific brackets. From a practical standpoint, the ideal approach uses age-specific 10-year brackets, including CN, irrespective of amyloid status, as this covers the greatest age range. Using this approach, the greatest accuracy was achieved using unsmoothed data and a Z-threshold of −3.5, with a cut-off of 98.5 subthreshold-voxels for total GM, 3.5 subthreshold-voxels in medial temporal GM, 29 subthreshold-voxels in frontal GM, 19.5 subthreshold-voxels in temporal GM, 24 subthreshold-voxels in the parietal GM, and 6 subthreshold-voxels in the occipital GM. Similar AUC values were observed when using a 2 mm smoothing kernel and a Z-threshold of −3.5, with a cut-off of 61.5 subthreshold voxels for total GM, 0.5 voxels in MTL GM, 13.5 subthreshold-voxels in frontal GM, 6 subthreshold-voxels in temporal GM, 14.5 subthreshold-voxels in the parietal GM, and 3.5 subthreshold-voxels in occipital GM.

### 3.6 Group differences in the validation sample

The observations regarding the reference group type (standard vs. age-specific) and the optimal Z-thresholds and smoothing kernels were validated in an independent in-house sample of 19 CN and 19 MCI patients (Table 6). The in-house sample was tested using 10-year brackets to allow the inclusion of the maximum number of participants. The standard reference group approach did not detect significant differences in the number of subthreshold voxels (p < 0.0001) between CN and MCI. Using the age-specific reference group approach, significant differences between the diagnostic groups were seen in medial temporal GM and temporal GM without smoothing and 2 mm smoothing at all Z-thresholds and at a Z-threshold of −4.5 in total GM, while the frontal and parietal GM showed trends toward a group difference.

**Table 6:** Number of subthreshold voxels for CN and MCI determined using an age-specific and standard reference approach in the validation sample.

Standard approach (reference group of 141 CN)																	
Smoothing kernel		0 mm				2 mm				4 mm				8 mm			
Group		CN		MCI		CN		MCI		CN		MCI		CN		MCI	
ROI	Z	Mean	SD	Mean	SD	Mean	SD	Mean	SD	Mean	SD	Mean	SD	Mean	SD	Mean	SD
Total GM	−2.5	1585	640	2863	1996	1160	639	2607	2333	725	769	2619	3278	438	1033	3431	6240
	−3.5	136	61	304	335	68	54	231	342	23	50	210	406	8	29	224	590
	−4.5	11	9	29	55	4	9	18	54	2	7	20	70	0	0	10	35
MTL	−2.5	84	58	320	409	64	62	311	457	51	73	333	574	24	54	404	793
	−3.5	12	13	54	93	8	14	44	97	5	14	33	92	1	4	26	88
	−4.5	2	7	11	26	2	7	9	28	2	7	8	29	0	0	2	7
Frontal cortex	−2.5	498	161	815	493	364	149	742	608	174	104	757	988	37	70	1152	2736
	−3.5	47	19	71	51	21	14	53	55	4	6	46	64	0	1	23	70
	−4.5	5	4	5	4	2	3	1	2	0	0	1	2	0	0	0	0
Temporal cortex	−2.5	303	106	715	634	190	99	661	727	114	112	762	1061	52	95	1241	2009
	−3.5	30	16	93	114	14	15	68	116	5	14	56	126	0	0	113	310
	−4.5	5	7	14	27	2	7	8	25	2	7	7	26	0	0	3	10
Parietal cortex	−2.5	256	156	477	340	219	180	505	462	159	175	568	747	54	77	682	1337
	−3.5	18	22	51	71	11	20	53	94	6	15	74	165	1	4	68	207
	−4.5	0	1	6	18	0	0	7	24	0	0	10	40	0	0	6	26
Occipital cortex	−2.5	106	55	205	121	81	41	176	140	41	33	152	237	12	25	130	314
	−3.5	6	5	13	13	2	3	10	20	0	0	12	42	0	0	19	78
	−4.5	0	0	0	0	0	0	0	1	0	0	1	4	0	0	0	2
10-year brackets (age-specific reference groups of 20 CN all within 5 years of the participant)																	
Smoothing kernel		0 mm				2 mm				4 mm				8 mm			
Group		CN		MCI		CN		MCI		CN		MCI		CN		MCI	
ROI	Z	Mean	SD	Mean	SD	Mean	SD	Mean	SD	Mean	SD	Mean	SD	Mean	SD	Mean	SD
Total GM	−2.5	4704	1613	12,012	7763	4300	2218	13,180	9424	4123	3298	8241	7505	4285	5068	11,016	12,187
	−3.5	871	375	3358	2715	676	440	3462	3047	552	629	1771	2665	560	896	2709	4981
	−4.5	**173**	**91**	**1059**	**979**	107	83	1018	1003	68	78	444	957	49	90	872	2483
MTL	−2.5	**186**	**172**	**1704**	**1024**	**153**	**192**	**1878**	**1208**	124	210	1496	1662	90	231	2010	2346
	−3.5	**37**	**43**	**590**	**491**	**27**	**43**	**627**	**575**	21	44	482	775	10	35	753	1383
	−4.5	**7**	**11**	**214**	**216**	**5**	**8**	**210**	**237**	2	6	143	270	0	0	294	640
Frontal cortex	−2.5	1212	319	2580	1992	1063	469	2858	2695	940	834	1305	1530	991	1664	1541	2483
	−3.5	214	65	543	504	145	71	521	607	86	111	174	247	72	214	220	446
	−4.5	44	19	120	128	21	14	94	130	8	16	23	40	7	21	27	70
Temporal cortex	−2.5	**820**	**311**	**4366**	**3161**	**674**	**390**	**4972**	**3916**	622	549	3315	4529	657	1038	5064	7264
	−3.5	**149**	**86**	**1565**	**1468**	**94**	**88**	**1728**	**1757**	74	120	982	2216	88	233	1766	4374
	−4.5	**30**	**23**	**611**	**647**	**15**	**16**	**628**	**715**	8	14	313	896	7	27	759	2404
Parietal cortex	−2.5	749	269	1488	935	695	347	1604	1074	598	473	1050	1001	419	643	1239	1744
	−3.5	130	69	306	222	101	69	293	228	69	93	178	307	46	163	199	522
	−4.5	24	15	68	56	15	13	56	59	10	18	41	108	9	38	40	147
Occipital cortex	−2.5	457	150	727	463	405	185	750	545	388	324	495	458	467	699	645	1072
	−3.5	63	27	128	87	45	29	109	91	28	37	46	62	49	115	63	152
	−4.5	10	5	23	19	5	4	16	14	2	3	6	11	5	16	6	23

Group differences were computed as Wilcoxon-Tests. Bold font and gray background indicate a significant group difference at p < 0.0001. CN = cognitively normal, MCI = mild cognitive impairment, ROI = region of interest, Z = Z-threshold, SD = standard deviation, GM = gray matter, MTL = medial temporal lobe. The smoothing kernel is reported in mm of full width at half-maximum.

### 3.7 Classification accuracy in the validation sample

The greatest AUC achieved using the standard reference group in this sample was 0.773 in the MTL without smoothing and in total GM with 8 mm smoothing (Table 7).

**Table 7:** Areas under the curve for comparing MCI and CN, determined using an age-specific and standard reference approach in the validation sample.

ROI = region of interest, AUC = area under the curve, Z = Z-threshold, GM = gray matter, MTL = medial temporal lobe, CN = cognitively normal. The smoothing kernel is reported in mm full width at half-maximum.

The greatest classification accuracies using the age-specific approach with 10-year brackets were achieved for medial temporal and temporal GM with no or 2 mm smoothing. The maximum was an AUC of 0.985 at a Z-threshold of −3.5 without smoothing in the MTL. Moderate AUC values ranging between 0.839 and 0.881 were observed for total GM without and with 2 mm smoothing. AUC values, especially in frontal and parietal, but also total GM were much lower in this sample than in the ADNI sample.

Using the age-specific 10-year brackets, the best classification accuracy was achieved using unsmoothed data and a Z-threshold of −3.5, with an extent cut-off of 1111.5 subthreshold-voxels for total GM, 161 subthreshold-voxels in MTL GM, 280 subthreshold-voxels in frontal GM, 294 subthreshold-voxels in temporal GM, 280 subthreshold-voxels in the parietal GM, and 64 subthreshold-voxels in the occipital GM. Similar AUC values were observed when using a 2 mm smoothing kernel and a Z-threshold of −3.5 with a cut-off of 1006.5 subthreshold voxels for total GM, 76.5 voxels in the MTL, 239 subthreshold-voxels in frontal GM, 243.5 subthreshold-voxels in temporal GM, 147 subthreshold-voxels in the parietal GM, and 48.5 subthreshold-voxels in occipital GM.

## 4 Discussion

We demonstrate that VBM could differentiate between MCI-like atrophy and atrophy in cognitively normal aging with very high accuracy. Age-specific reference groups significantly increased accuracy, more so than regression-based approaches and using amyloid-negative reference groups. Constraining the age range for the reference template to within five years of the patients’ ages (i.e., a 10-year bracket centered on the patient’s age) improved accuracy substantially. A further narrowing of the age range led to marginal accuracy improvement only while reducing the number of patients that could be examined.

### 4.1 Age-adjustment outweighs amyloid-status

Approaches accounting for age differences within the reference group consistently outperformed those that merely used a reference group in a similar age range as the patients. This finding is likely because the ages of MCI patients examined often range from 60 to 90 and beyond [Hedderich et al., 2022, Hirata et al., 2005, Matsuda et al., 2012, Waragai et al., 2014]. A considerable loss of brain volume characterizes this part of the life span [Bethlehem et al., 2022, Hedman et al., 2012], leading to an overestimation of atrophy in patients at the upper end of the age range and an underestimation in patients at the younger end.

5-year age brackets did not perform better than 10-year brackets, likely because the width of the bracket defined the maximum age difference between the patient and the members of the reference sample for that age. For most ages, especially near the middle of the age range, there were so many CNs within the age brackets that the 20 closest CN to the patient’s age were much closer to the patient’s age than the width of the bracket suggested.

Accounting for age using W-scores improved classification accuracy but not to the degree achieved using age-specific reference groups (for details please see the Supplementary Materials). While the W-scores account for age differences, they are based on the assumption of a strictly linear relationship between age and GM volume. There is evidence, however, that the rate of atrophy in some brain regions does not follow a linear trajectory [Bethlehem et al., 2022, Fjell et al., 2013, Hedman et al., 2012, Pfefferbaum et al., 2013, Scahill et al., 2003] and may even accelerate with increasing age, whereas it has also been reported that it levels off around the age of 80 in CN [Schuff et al., 2012]. Thus, the age-specific brackets likely captured age-related changes in GM volume more accurately.

Contrary to our hypothesis, data indicated that an amyloid-negative reference group was only superior to a mixed one when not accounting for age. The most parsimonious explanation for this finding is that the age effects on GM volume outweigh those of amyloid-positivity. Previous work indicated that differences in GM volume between amyloid-negative and amyloid-positive CN are detectable but subtle [Becker et al., 2011, Harrison et al., 2021, Whitwell et al., 2013]. Furthermore, amyloid-positive CN only made up 24% of the CN reference sample, limiting the influence of amyloid-positivity. Another possible explanation for the fact that removing amyloid-positive CN from the reference groups increased classification accuracy when age was not accounted for is that amyloid-positive CNs in the present sample were older than their amyloid-negative counterparts and MCI patients. Their removal thus moved the average age of the reference groups closer to that of the patients. In summary, our data suggest that the amyloid status in a reference sample is negligible if appropriate measures account for age effects.

### 4.2 Achieved level of accuracy

The highest accuracy in differentiating between CN and MCI in the ADNI sample was a perfect 1 when using age-specific reference groups. The AUC previously reported for the distinction between CN and MCI ranged between 0.86 and 0.949 [Hirata et al., 2005, Matsuda et al., 2012, Waragai et al., 2014]. However, Z-statistics were computed using the whole control group's means and standard deviations in those studies. The Z-statistics using the mean and SD of all CN were much lower, with the highest AUC being 0.825. A possible explanation may be that the MCI patients in the present ADNI sample were much more mildly impaired, with an average MMST of 28.37, while MCI patients in the other studies had average MMSEs ranging between 26 and 27 [Hirata et al., 2005, Matsuda et al., 2012, Waragai et al., 2014].

It is conceivable that the values we observed were particularly high since the reference groups for the computation of the Z- and W-statistics were derived from the same sample. However, in comparing the results to the literature, it has to be considered that most previous studies have taken a similar approach [Hirata et al., 2005, Matsuda et al., 2012, Waragai et al., 2014]. Furthermore, in our independent validation sample, we also achieved very high levels of classification accuracy with AUC up to 0.985 when using age-specific reference brackets, while the conventional approach using the mean and SD of the whole CN group only reached a maximum AUC of 0.773.

### 4.3 Differences in atrophy patterns

In the ADNI sample, the highest classification accuracy was observed for cortical areas, with the poorest performance observed for the MTL. In the validation sample, in line with the literature [Hirata et al., 2005, Matsuda et al., 2012, Waragai et al., 2014], the greatest AUCs were observed in the temporal lobe, especially the MTL. A possible explanation is that only about half of the MCI patients in the ADNI sample were amyloid-positive, indicating AD pathology. The number of patients exhibiting AD-typical temporal lobe atrophy [Scheltens et al., 1992] in this group would thus be expected to be lower than in the validation sample, where all but one participant had biomarkers indicative of AD pathology.

Another explanation could be that MCI in the validation sample was defined solely based on logical memory. In the ADNI sample, however, MCI was defined by impairment in logical memory, but also a score of 0.5 on the Clinical Dementia Scale Sum of Boxes (CDR-SB), which can also be caused by mild impairments in other cognitive domains, not as strictly associated with the MTL as memory [Balsis et al., 2015, Cedarbaum et al., 2013].

### 4.4 Optimal smoothing kernels and thresholds for classification

Independent of the type of reference groups used, the best classification accuracy was achieved without spatial smoothing or with a small smoothing kernel of 2 mm at FWHM, in line with a study distinguishing between CN and AD patients using Z-statistics derived from GM data (Komatsu et al., 2018). Generally, in group comparisons using VBM data, smaller smoothing kernels are more sensitive [Shen and Sterr, 2013], but larger smoothing kernels perform better in small samples [Mikl et al., 2008, Shen and Sterr, 2013]. However, the present data indicate that the concept of ‘larger kernels for smaller samples’ does not apply when comparing an individual to a group average. Arguably, this is because the current approach does not rely on the spatial overlap of atrophy between patients, as the presence of only one ‘patient’ eliminates the averaging of atrophy.

In the literature on voxel-wise analyses of GM atrophy, cut-offs for Z-statistics indicating significant atrophy tend to be around −2 [Matsuda et al., 2012, Matsuda, 2016] or −2.5 [Caspers et al., 2021]. Our data indicate that a higher classification accuracy may be achieved using lower thresholds with an optimum around Z = -3.5. However, this analysis will fail if the threshold is lowered too far, as no more subthreshold voxels are detected.

### 4.5 Limitations

The main limitation of this study lies in the fact that the CN sample used to generate the reference groups was also used in subsequent classification analyses. This procedure is common in the literature [Hirata et al., 2005, Matsuda et al., 2012] but may have inflated the classification accuracy within the ADNI sample. The approach was chosen despite this limitation to ensure that the definition of the amyloid status was consistent across participants. This is also why only ADNI participants with an amyloid PET within a year of the MR scan were included, even though this limited the sample size.

Even though we were able to reproduce our findings from the ADNI data in a separate sample using a different 3 T MR scanner, it needs to be taken into consideration, that this sample was relatively small consisting of only 19 participants in each group.

To validate our approach, we tested it in an independent validation sample and observed very high but slightly lower classification accuracy levels than in the ADNI sample. Furthermore, the optimum number of subthreshold voxels, i.e., the extent of atrophy that best differentiated between CN and MCI, was much lower in the ADNI sample than in the validation sample. A possible explanation may be that the MCI patients in the validation sample were more severely affected (average MMSE of 25.79) than the MCI patients in the ADNI sample (average MMSE of 28.37). It is also conceivable that the number of subthreshold voxels that best discriminated between the groups was so low in the ADNI sample because that sample was used to generate a reference group, as highlighted above. Consequently, extent thresholds would presently have to be specifically defined for different samples or centers. However, further research may identify factors that contribute to inter-site variability in thresholds. By comparing measurements from a number of different sites, it also may be possible to identify thresholds that reduce accuracy to a tolerable level, but result in the greatest consistency across sites and samples. The use of larger databases that do not incorporate amyloid-status may in turn allow the generation of larger, more representative reference groups.

## 5 Conclusions

We expanded on the established method of using VBM-based Z-statistics to quantify GM atrophy systematically. We observed that the accuracy with which MCI-like atrophy can be distinguished from age-related atrophy could be substantially increased using age-specific reference groups. In contrast, limiting reference groups to amyloid-negative CN did not improve diagnostic accuracy.

## CRediT authorship contribution statement

**Nils Richter:** Conceptualization, Methodology, Software, Formal analysis, Writing – original draft. **Stefanie Brand:** Data curation, Formal analysis. **Nils Nellessen:** Methodology, Software. **Julian Dronse:** Investigation, Data curation, Writing – review & editing. **Hannes Gramespacher:** Data curation, Writing – review & editing. **Maximilian H.T. Schmieschek:** Data curation, Methodology, Writing – review & editing. **Gereon R. Fink:** Writing – review & editing, Resources, Funding acquisition. **Juraj Kukolja:** Conceptualization, Supervision, Writing – review & editing, Resources, Funding acquisition. **Oezguer A. Onur:** Supervision, Investigation, Writing – review & editing, Resources, Funding acquisition.

## Declaration of Competing Interest

The authors declare that they have no known competing financial interests or personal relationships that could have appeared to influence the work reported in this paper.

## Data Availability

Data will be made available on request.
