# Supplementary material for: Fine-grained age-matching improves atrophy-based detection of mild cognitive impairment more than amyloid-negative reference subjects
Source: Neuroimage Clin. 2023 Sep 9;40:103508. doi: 10.1016/j.nicl.2023.103508 (PMC10514218; doi:10.1016/j.nicl.2023.103508)
Supplement: Supplementary data 1 [file mmc1.docx]

**Supplementary Materials**

*W-Scores*

W-scores are similar to Z-scores but adjusted for a specific covariate using linear regression [Iaccarino et al., 2021; Jack et al., 1997; La Joie et al., 2012]. In the present study, that covariate was age. Hence, voxel-wise regressions were computed between age and GM maps in the CN group. Voxel-wise W-scores were then obtained for each participant by subtracting the GM value expected for the participants’ age based on the linear regression (parameter estimate for GM plus the parameter estimate for age multiplied by age) from the participants’ GM value and dividing this number by the standard deviation of the residuals in the control group (for a detailed explanation see [La Joie et al., 2012]. W-scores were computed in two ways: (1) using all 141 CN from the ADNI sample irrespective of amyloid status to perform the linear regression and (2) using only the 97 amyloid-negative CN.

*Classification accuracy using W-scores*

Using W-scores by computing a linear regression between age and GM across amyloid-negative and -positive CN from the ADNI sample (n = 141), the highest accuracies were seen with no or 2 mm smoothing at Z-thresholds of -3.5 and -4.5. The highest AUC was 0.778 in total GM, followed by 0.775 for frontal GM. Accuracies did not differ when generating the W-scores based only on amyloid-negative controls (n = 97) with a maximum AUC of 0.794 in frontal GM without smoothing at a Z-threshold of -3.5 (**Supplementary Table 1**).

**Supplementary Table 1.** Areas under the curve for comparing MCI and CN, using W-scores derived from all CN and amyloid-negative CN only.

| Reference type | | W-score based on all CN (141) | | | | W-score based on amyloid-negative CN (97) | | | |
| --- | --- | --- | --- | --- | --- | --- | --- | --- | --- |
| Smoothing | | 0mm | 2mm | 4mm | 8mm | 0mm | 2mm | 4mm | 8mm |
| ROI | Z | AUC | AUC | AUC | AUC | AUC | AUC | AUC | AUC |
| Total GM | -2.5 | 0.676 | 0.671 | 0.582 | 0.528 | 0.667 | 0.67 | 0.58 | 0.526 |
|  | -3.5 | 0.729 | 0.715 | 0.63 | 0.557 | 0.742 | 0.714 | 0.632 | 0.555 |
|  | -4.5 | 0.778 | 0.744 | 0.666 | 0.596 | 0.771 | 0.754 | 0.671 | 0.596 |
| MTL | -2.5 | 0.649 | 0.645 | 0.588 | 0.556 | 0.643 | 0.643 | 0.585 | 0.553 |
|  | -3.5 | 0.655 | 0.67 | 0.631 | 0.578 | 0.667 | 0.673 | 0.626 | 0.576 |
|  | -4.5 | 0.65 | 0.668 | 0.657 | 0.601 | 0.65 | 0.697 | 0.652 | 0.597 |
| Frontal cortex | -2.5 | 0.725 | 0.693 | 0.573 | 0.531 | 0.73 | 0.698 | 0.581 | 0.536 |
|  | -3.5 | 0.775 | 0.751 | 0.621 | 0.548 | 0.794 | 0.764 | 0.627 | 0.555 |
|  | -4.5 | 0.76 | 0.747 | 0.662 | 0.575 | 0.734 | 0.745 | 0.67 | 0.586 |
| Temporal cortex | -2.5 | 0.663 | 0.648 | 0.568 | 0.531 | 0.662 | 0.642 | 0.561 | 0.527 |
|  | -3.5 | 0.705 | 0.692 | 0.606 | 0.544 | 0.711 | 0.687 | 0.601 | 0.538 |
|  | -4.5 | 0.7 | 0.678 | 0.65 | 0.567 | 0.714 | 0.705 | 0.648 | 0.562 |
| Parietal cortex | -2.5 | 0.674 | 0.661 | 0.534 | 0.515 | 0.665 | 0.653 | 0.532 | 0.518 |
|  | -3.5 | 0.724 | 0.704 | 0.585 | 0.506 | 0.722 | 0.691 | 0.585 | 0.504 |
|  | -4.5 | 0.681 | 0.693 | 0.632 | 0.544 | 0.625 | 0.67 | 0.63 | 0.543 |
| Occipital cortex | -2.5 | 0.662 | 0.644 | 0.555 | 0.504 | 0.657 | 0.637 | 0.555 | 0.506 |
|  | -3.5 | 0.707 | 0.68 | 0.606 | 0.519 | 0.708 | 0.668 | 0.608 | 0.517 |
|  | -4.5 | 0.635 | 0.7 | 0.648 | 0.568 | 0.598 | 0.686 | 0.64 | 0.566 |

ROI = region of interest, Z = Z-threshold, GM = gray matter, MTL = medial temporal lobe, CN = cognitively normal. The smoothing kernel is reported in mm full width at half-maximum.

**References**

Iaccarino L, La Joie R, Edwards L, Strom A, Schonhaut DR, Ossenkoppele R, Pham J, Mellinger T, Janabi M, Baker SL, Soleimani-Meigooni D, Rosen HJ, Miller BL, Jagust WJ, Rabinovici GD (2021): Spatial Relationships between Molecular Pathology and Neurodegeneration in the Alzheimer’s Disease Continuum. Cerebral Cortex 31:1–14.

Jack CR, Petersen RC, Xu YC, Waring SC, O’Brien PC, Tangalos EG, Smith GE, Ivnik RJ, Kokmen E (1997): Medial temporal atrophy on MRI in normal aging and very mild Alzheimer’s disease. Neurology 49:786–794.

La Joie R, Perrotin A, Barre L, Hommet C, Mezenge F, Ibazizene M, Camus V, Abbas A, Landeau B, Guilloteau D, de La Sayette V, Eustache F, Desgranges B, Chetelat G (2012): Region-Specific Hierarchy between Atrophy, Hypometabolism, and -Amyloid (A ) Load in Alzheimer’s Disease Dementia. Journal of Neuroscience 32:16265–16273.
